# Photocatalytic Degradation of 4,4′-Isopropylidenebis(2,6-dibromophenol) on Sulfur-Doped Nano TiO_2_

**DOI:** 10.3390/ma15010361

**Published:** 2022-01-04

**Authors:** Joanna B. Kisała, Gerald Hörner, Adriana Barylyak, Dariusz Pogocki, Yaroslav Bobitski

**Affiliations:** 1 College of Natural Sciences, University of Rzeszow, Pigonia 1 Str., 35-310 Rzeszow, Poland; d.pogocki@ichtj.waw.pl (D.P.); yaroslav.v.bobytskyi@lpnu.ua (Y.B.); 2 Inorganic Chemistry IV, University of Bayreuth, Universitätsstrasse 30, D-95440 Bayreuth, Germany; gerald.hoerner@uni-bayreuth.de; 3 Department of Therapeutic Dentistry, Danylo Halitsky Lviv National Medicinal University, Pekarska Str. 69, 79010 Lviv, Ukraine; adriana.barylyak5@gmail.com; 4 Institute of Nuclear Chemistry and Technology, Dorodna 16, 03-195 Warsaw, Poland; 5 Department of Photonics, Lviv Polytechnic National University, S. Bandera Str. 12, 79013 Lviv, Ukraine

**Keywords:** S-doped nano-TiO_2_, photocatalysis, TBBPA, persistent organic pollutants

## Abstract

In present work, we examine the photocatalytic properties of S-doped TiO_2_ (S1, S2) compared to bare TiO_2_ (S0) in present work. The photocatalytic tests were performed in alkaline aqueous solutions (pH = 10) of three differently substituted phenols (phenol (I), 4,4′-isopropylidenebisphenol (II), and 4,4′-isopropylidenebis(2,6-dibromophenol) (III)). The activity of the catalysts was evaluated by monitoring I, II, III degradation in the reaction mixture. The physicochemical properties (particle size, ζ-potential, E_bg_, Eu, E^0^_cb_, E^0^_vb_, σ_o_, K_L_) of the catalysts were established, and we demonstrated their influence on degradation reaction kinetics. Substrate degradation rates are consistent with first-order kinetics. The apparent conversion constants of the tested compounds (k_app_) in all cases reveal the sulfur-loaded catalyst S2 to show the best photocatalytic activity (for compound I and II S1 and S2 are similarly effective). The different efficiency of photocatalytic degradation I, II and III can be explained by the interactions between the catalyst and the substrate solution. The presence of bromine substituents in the benzene ring additionally allows reduction reactions. The yield of bromide ion release in the degradation reaction III corresponds to the Langmuir constant. The mixed oxidation-reduction degradation mechanism results in higher degradation efficiency. In general, the presence of sulfur atoms in the catalyst network improves the degradation efficiency, but too much sulfur is not desired for the reduction pathway.

## 1. Introduction

The depletion of water resources is one of the key problems of the modern world. The overuse of water and its growing pollution has caused the scarcity of water resources. The contamination of industrial wastewater has become a major issue due to the tremendous diversity of potential micro-pollutants originating from industrial products such as plastics, agrochemicals, pharmaceuticals, or household chemicals, which can seep into the ground and surface waters. Among the burdensome pollutants, aromatic hydrocarbons are one of the most toxic, but still utilised widely in petrochemical, chemical, and pharmaceutical industries. The presence of aromatic compounds in aqueous environments creates serious problems due to complex mechanisms of their toxicity, persistence in the environment, and significant bioaccumulation. The increasing public concern with these environmental pollutants brings upon the need to develop novel treatment methods, in which solar-light-induced photocatalytic degradation of micro-pollutants gains growing attention. The utilisation of colloidal semiconductors and introducing catalysts to promote specific redox processes on semiconductor surfaces were developed in the last decades.

One way to increase the overall yield of reactive species formed on the illuminated surface of a semiconducting material is to grow the number of the absorbed solar-light quanta. This goal can be achieved by the formation of new spots in the material, which are able to absorb more light from the visible part of the solar-light spectrum [1]. The spots of locally narrowed band gap may be formed by an incorporation of metal or non-metal dopants into the TiO_2_ lattice. Doping is actually the most common strategy of the TiO_2_ band gap engineering aimed to enhance its photocatalytic activity; such modification allows the TiO_2_-based material to harvest some part of the visible-light spectrum.

Among non-metal main group elements, recent scientific interest has focused mainly on doping TiO_2_ with nitrogen [2,3,4,5], carbon [5,6,7,8], and sulfur [9,10,11,12,13,14,15]. Some dopants such as nitrogen and carbon are incorporated as anions replacing oxygen in the lattice of TiO_2_. In contrast, sulfur can be incorporated in cationic and anionic formulations, depending on the applied synthesis method. Sulfur-doping creates localised sulfur electronic-states within the TiO_2_ band gap. The electron promotion from these localised states would be responsible to its visible light absorption [11,16]. The group of Ohno and other groups [9,17] have found that, when taking thiourea or sulphate as sulfur sources of the S-doped TiO_2_ nanopowders, sulfur atoms are incorporated as cations, replacing Ti ions. They have provided additional evidence that, due to the better compatibility of ionic radii, the substitution of Ti^4+^ by S^6+^ is thermodynamically more favourable than the replacement of O^2−^ by S^2−^. The substitution of Ti^4+^ by S^6+^ induces sulfur 3 s states lying just above the oxygen 2p valence states, and S 3p states contributed to the conduction band (CB) of TiO_2_ [13]. The other reports [18,19] also notice presence of two ionic forms of sulfur in the TiO_2_ nanoparticles when thiourea was a sulfur source. The S and C doping induced narrowing of the effective TiO_2_ band gap was caused by the formation of additional dopant related mixed electronic states inside the anatase band gap, reducing the electron transition energy, and allowing visible-light induced photo excitation [20]. Simultaneously, the co-doped carbon may form carbonaceous species on the surface of TiO_2_, which acts as a photo-sensitizers like organic dyes [8].

TiO_2_ seems to be an almost ideal photocatalyst for photocatalytic degradation organic compounds. The photogenerated holes are highly oxidizing, whereas photogenerated electrons are reducing enough to produce superoxide from dioxygen. It is suggested that oxidative and reductive photocatalytic reactions take place simultaneously on TiO_2_ particles. In a photocatalytic process, the generated electron-hole pairs must be trapped to avoid recombination. The electron and hole may then migrate to the catalyst surface where they participate in redox reactions. Water or hydroxyl ion (OH^−^) is the possible trap for the hole (h^+^), leading to the formation of ^•^OH. It is well known that O_2_ is essential for the photocatalytic oxidation of organic compounds. Its presence depresses the recombination of electron-hole pairs. Oxygen close to the TiO_2_ surface may trap the electron (e^−^) to generate the superoxide radical anion (O_2_^•−^), which is unstable, reactive and evolves into additional ^•^OH [21]. Much research is focused on the hole-initiated oxidative degradation of organic pollutants. For aqueous suspensions of TiO_2_, hydroxyl radicals are the dominant reactive species responsible for the photocatalytic oxidation of phenols. 

In this paper, we report photocatalytic activities of TiO_2_ doped with sulfur. Subsidizing with sulfur allowed for tuning of the photocatalytic properties of TiO_2_ via extension of the radiation absorption range, as well as a change in the redox potentials of the catalyst. The efficacy of the photocatalysts were benchmarked in degradation experiments with a selection of three phenols: parent phenol (I), bisphenol-A (II), and 3,3′,5,5′-tetrabromobisphenol-A (III), are common environmental pollutants. Our interest was to investigate the degradation kinetics of chosen phenols and to correlate it with the photocatalysts’ properties. The new catalysts were indeed found superior to commercially available TiO_2_. Knowledge of the interaction of the catalyst with an organic compound will increase the photocatalytic efficiency of POPs degradation.

## 2. Materials and Methods

### 2.1. Chemicals

TiO_2_ 325 mesh, 95% (S0) was obtained from Aldrich and used as received, whereas SC doped catalysts (S1, S2) have been prepared according to the routines reported by Ivanov et al. [22]. SC doped nanopowders were synthesized in a solid-phase method where metatitanic acid (MA) and thiourea (TU) were triturated in an agate mortar to obtain a homogeneous mass, further annealed in an air atmosphere at 500 °C for 1 h. S1 was obtained in 1:0.44, and S2 1:1.34 molar ratio of MA:TU. 4,4′-Isopropylidenebis(2,6-dibromophenol) (TBBPA) (97%) was purchased from Alfa Aesar, 4,4′-isopropylidenebisphenol (BPA) (>98%) from Sigma-Aldrich, and phenol from Riedel-de-Haen (99%). Other chemicals were purchased at highest available purity and used as received.

### 2.2. Photocatalysts Characterization

The hydrodynamic diameter of semiconductor particles and the electrokinetic potential (zeta potential, ζ potential) [23] were measured as a colloidal dispersion with a concentration of 6.25 × 10^−4^ g cm^−1^ at pH = 10. Zeta potentials were determined by electrophoretic mobility measurement in a particle suspension. The hydrodynamic diameter of the catalysts was measured by Dynamic Light Scattering (DLS). These measurements were performed with NanoPlus 3 HD analyser (Particulate Systems, Micromeritics, Norcross, GA 30093, USA) following the procedure explained in the application note No. 029 [24].

The surface area of the catalysts was determined by ASAP 2020 Accelerated Surface Area and Porosimetry (Micromeritics, Norcross, GA 30093, USA).

The phase identification of doped TiO_2_ was carried out by powder X-ray diffraction, using the D8 Advance (BRUKER) diffractometer in the reflection mode with Cu target Kα radiation. The average crystallite sizes (D) of TiO_2_ samples were calculated from the powder XRD line widths by applying the Debye–Scherrer Equation (1) [25,26]

(1) $$D=\frac{0.89\lambda }{\mathrm{Bcos}\Theta }$$

where λ is the wavelength of the X-ray in nanometres, B is the width at half peak-height in radian, and θ is the angle between the incident and diffracted beams in angular degrees.

The optical measurements of the catalysts were carried out using Agilent Technologies Cary Series UV–Vis–NIR Spectrophotometer in the wavelength range from 180 to 1200 nm. The UV-Vis spectra of organic compounds solutions were measured on a VWR UV-VIS 3100 PC spectrophotometer. The morphology and particles size of prepared nanoparticles were evaluated by a scanning electron microscope (SEM) and transmission electron microscope (TEM). The specimen was subject to examination with the use of VEGA XMH scanning electron microscope (SEM, Tescan, Brno, Czech Republic) equipped with an energy dispersive X-ray spectrometer (EDS, Oxford Instruments, Abingdon, UK). The JEOL-JEM-1011 TEM microscope was operated at an accelerating voltage of 80 kV with a resolution of 0.2 nm was used. The powder samples were prepared by air-drying a drop of a sonicated suspension onto copper grids.

Experiments of dark adsorption-desorption of phenols on catalysts were carried out in 20 cm^3^ vials equipped with a magnetic stirring bar. The samples of phenols with catalysts were prepared by mixing the phenol solutions with the solid dispersion. The exposure to light was minimised. The samples at pH = 10 were all sealed and kept 60 min in the dark, to establish adsorption equilibrium. The concentration of phenols ranged from 2.003 × 10^−4^ to 2.96 × 10^−3^ mol dm^−3^ and the catalysts’ concentration from 6.45 × 10^−4^ − 6.75 × 10^−4^ g cm^−3^. The solution pH was adjusted to the value of 10 ± 0.1 using 2 mol dm^−3^ NaOH solution. Adsorption was followed by HPLC measurement of equilibrium phenols concentration in the aqueous phase.

Determination of surface charge was performed by the potentiometric acid-base titration procedure was as follows: To a volume of 50 mL of a blank solution or aqueous catalyst suspensions (S0, S1, S2 catalyst concentrations, respectively: 6.74 × 10^−4^; 6.34 × 10^−4^; 6.54 × 10^−4^ g dm^−3^) was added 5.00 × 10^−2^ cm^3^ of 60% HClO_4_ (9.56 × 10^−3^ mol dm^−3^) for stabilisation of ionic strength of mixtures. The NaOH titrant solution (0.1 mol dm^−3^) was added as 5 × 10^−1^ ± 0.02 cm^3^ increment until the pH and the potentials of the mixtures became constant. The direct measurement of pH was obtained after the calibration of the apparatus in the standard buffers of pH 2; 4; 7; 10. 

### 2.3. Photocatalytic Degradation of Aromatic Compounds

**Chart 1:**
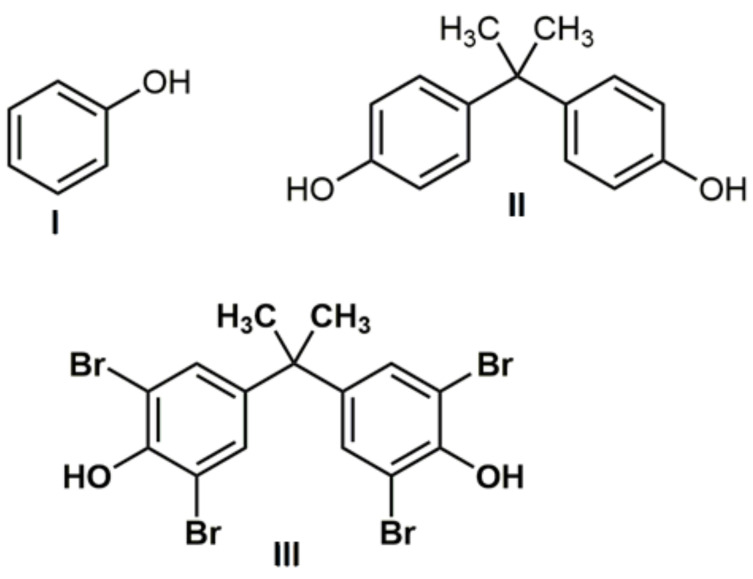
I—Phenol, II—4,4′-isopropylidenebisphenol (BPA); III—4,4′-Isopropylidenebis(2,6-dibromophenol) (TBBPA).

The photocatalytic activities of the S-TiO_2_ photocatalysts were evaluated in degradation experiments with three water-soluble aromatic compounds: phenol (PhOH, Chart 1 I), 4,4′-isopropylidenebisphenol (BPA, Chart 1 II), and 4,4′-isopropylidenebis(2,6-dibromophenol) (TBBPA, Chart 1 III). In a typical experiment, an aqueous solution of **I**, II or III (750 cm^3^; [X = I, II or III] = 2.0 × 10^−4^ mol dm^−3^, with the pH adjusted to the value of 10 ± 0.1 using 2 mol dm^−3^ NaOH solution, next 0.07% *w*/*v* (0.67 g dm^−3^) of semiconductor-catalyst powder was dispersed. The suspension was stirred for 30 min in the dark. The photocatalytic degradation was performed using a Heraeus LRS2 photoreactor of 750 cm^3^ volume in open air condition. Illumination was provided by an excimer lamp TQ150 (150 Watt, with forced water cooling down to 25 °C, of ca. 47 W light energy flux integrated over the 200–600 nm range, of power density 4.696 mW cm^−2^ measured by digital lux meter Peak Tech 5025 that gives light intensity ca. 7.88 × 10^19^ photons per second) operated by utilising a vertically arranged immersion tube, immersed into the continuously stirred reaction suspension. The photocatalytic reaction was performed up to 120 min illumination-time. During the reaction, 5 cm^−3^ samples were collected from the reactor at regular time-intervals (from first to tenth minute every two minutes, then every ten minutes up to 2 h). After filtration, the concentration of the substrates was determined.

The decay of testing compounds concentration was monitored applying high performance liquid chromatography (HPLC, Shimadzu, Japan) equipped with a UV detector (SPD-10AV) and a C18 column (Knauer 250 mm × 4.6 mm with precolumn, Eurospher II, 100-5 C18 H). Mobile phase: 70% acetonitrile and 30% water, flow rate: 1.0 cm^3^ min^−1^, injection volume: 20 μL, absorbance detection: 275 nm.

Total solubilised bromide (Br¯) was determined potentiometrically using a multimeter (CPC 411, Elmetron, Poland) equipped with a bromide ion-selective electrode (EBr-01, Hydromet, Poland) with the silver chloride electrode (RL-100, Hydromet, Poland) as a reference electrode.

### 2.4. Computational Methods

The quantum-chemical calculations were performed at standard state (298.15 K; 105 Pa (1 atm)) applying the NWChem 7.0 quantum-chemical code [27]. The electronic energies (E°298), zero-point vibrational energies (ZPVE) and heat capacities, Cv(T)’s (10 ≤ T/K ≤ 298) for investigated species were calculated from vibration, translation, and external rotation contributions based on the vibration frequencies and structures obtained from the B3LYP/6-311++G(2d,2p). The People-style basis set was used as provided by the EMSL Basis Set Library [28].

## 3. Results and Discussion

### 3.1. Photocatalysts Characterization

**Figure 1:**
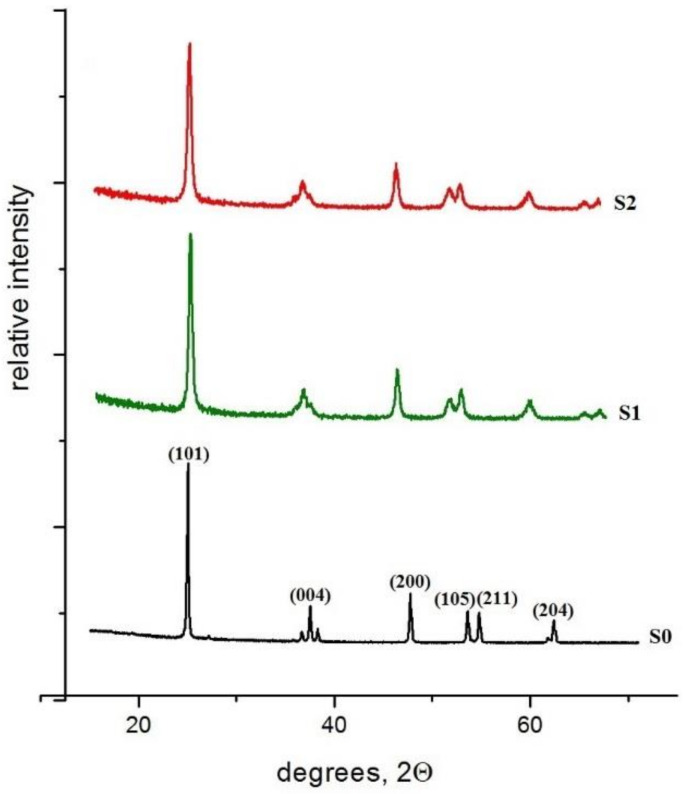
XRD pattern of catalysts, S1—green line, S2—red line; S0—black line.

The XRD patterns of S -doped TiO_2_, and commercial TiO_2_ are presented in Figure 1. Powder diffraction data revealed the prepared materials to have anatase structure (Figure 1). The average crystallite sizes of S0, S1, S2, are 46.0, 18.5, and 20.0 nm, respectively. 

**Figure 2:**
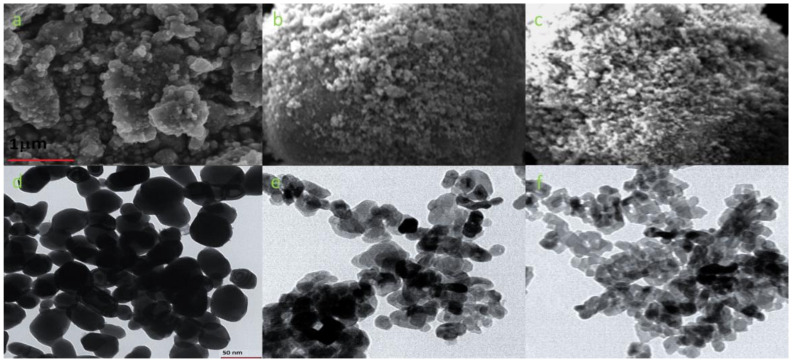
SEM images of S0 (**a**), S1 (**b**), S2 (**c**); TEM images of S0 (**d**), S1 (**e**), S2 (**f**).

The analysis of SEM images of the catalysts S1, S2 and S0 (Figure 2a–c) showed slight differences in the morphology of the tested materials. All materials consisted of aggregates of crystals. The aggregates S1 and S2 comprised ultrafine crystals, while the crystals of the aggregates S0 were larger. This is reflected in the specific surface area of the catalysts. The analysis of TEM images (Figure 2d–f) also showed differences in catalyst sizes. The TEM images show that the nanoparticles have a spherical form with a diameter of ca. 50 for S0, 25, and 15 nm for S1 and S2 respectively, including large agglomerates. The zeta potential dictated the sign and amount of the surface charge in relation to the surrounding conditions. The surface of TiO_2_ particles dispersed in water was covered by hydroxyl groups. Their ionisation equilibria could be written as [29]:

(2) $${\mathrm{TiOH}}_{2\;}^{+}⇄\mathrm{TiOH}+H^{+}\;K_{1}$$

(3) $$\mathrm{TiOH}⇄{\mathrm{TiO}}^{-}+H^{+}\;K_{2}$$

The pH at which the surface of the catalyst was neutral is the point of zero charge (pH_pzc_). At pHs lower than pH_pzc_, partial protonation of TiO_2_ led to positive surface charge and positive zeta potential. Values of pH higher than pH_pzc_ cause partial deprotonation of catalyst surface and led to negative surface charge. S-OH groups present in S1 and S2 underwent similar mechanisms of protonation/deprotonation leading to positive and negative surface charges; however, they were much more acidic, accounting for the higher zeta potential value (ξ-potential S2 > S1 > S0, Table 1). The measured values of ξ-potential for all samples were below −30 mV, indicating good stability of water suspensions of examined materials. 

As DLS measurements showed, the catalysts did not exist in their isolated primary particle size form, but as aggregates, hence particle size in water ca. 340–504 nm. The dispersity values of catalyst suspensions indicated that the samples were polydisperse (due to agglomeration). The hydrodynamic diameter rose with the increase in dispersity index. An enhancement in the absolute value of the zeta potential increased the electrostatic repulsion force, suppressed agglomeration, and then reduced the hydrodynamic size of the dispersion. The S0 particles diameter were found to be the smallest; interestingly, the average particle diameter increased with increase of sulfur content (Table 1).

Compared with commercial TiO_2_, the particle size of the S-doped TiO_2_ samples was much smaller, resulting in larger specific surface area. Small particle size can shorten the route for an electron migration from the interior of TiO_2_ to surface, which can reduce the recombination of h^+^ and e^−^. Moreover, the larger the surface area, the more active sites on the surface and the higher probability of substrate adsorption.

The surface of the oxide catalyst in an aqueous solution was covered with H^+^ and OH^−^ ions [29]. The surface charges of the catalysts were determined from potentiometric titration data using following equation:

(4) $$\sigma_{o}=\frac{F\left(C_{a}-C_{b}\right)+\left[{\mathrm{OH}}^{-}\right]-\left[H^{+}\right]}{m\;S}\;\left[\frac{C}{m^{2}}\right]$$

**Figure 3:**
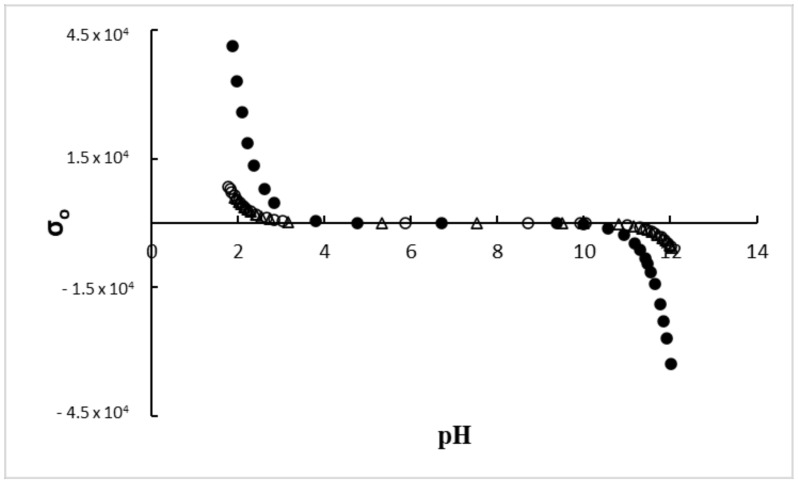
Surface charge density of studied catalysts. Filled circles (●) represent the S0-catalyst, the open circle (o) the S1-catalyst suspension, while open triangle (∆) the S2-catalyst suspensions.

Here, σ_o_ is the surface charge density (in C m^−2^), F is the Faraday constant (96,485.3 C mol^−1^); C_a_ and C_b_ are the concentrations (mol dm^−3^) of acid and base, measured after addition of catalysts to the solution; [OH^−^] and [H^+^] represent the adsorption densities of OH^−^ and H^+^, respectively, as measured from the pH of the solution; m and S refer to the mass (g) and surface area (m^2^ g^−1^) of catalysts, respectively. The pK_1_ and pK_2_ constants were determined from plots of σ_o_ = f(pH) for all catalysts (Figure 3). The acidity constants were used to calculate the pH_pzc_ of solids using the relationship:

(5) $${\mathrm{pH}}_{\mathrm{pzc}}=\frac{1}{2}\left({\mathrm{pK}}_{1}+{\mathrm{pK}}_{2}\right)$$

The surface charge density of the catalysts increases monotonously with the sulfur content of the samples along the series S0 < S1 < S2. These results agree with the results of the zeta potential measurements. Clearly, catalysts doped with sulfur are more acidic in nature as the pH_pzc_ values of powders (S0, S1, S2) increase with the dopant content (6.07, 6.84, 6.96, respectively, Table 2). We associate this trend with the introduction of strongly acidic sites on the surface [30]. The highest pH_pzc_ value for S2 indicates the highest number of acidic sites on the surface of this catalyst.

Phenols absorption on catalysts were analysed according to the Langmuir adsorption model. In this model, all adsorption sites had the same sorption activation energy. The Langmuir equation can be written as [31]:

(6) $$\frac{C_{e}}{q_{e}}=\frac{1}{Q_{\max}K_{L}}+\frac{C_{e}}{Q_{\max}}$$

where C_e_ is the concentration of adsorbate in the solution at equilibrium (g dm^−3^), q_e_ is the amount of phenol adsorbed per unit mass of catalyst (g g^−1^), Q_max_ is the maximum uptake per unit mass of catalyst (g g^−1^), representing the maximum monolayer capacity of adsorbent; it can also be interpreted as the total number of binding sites that are available for sorption. K_L_ is the Langmuir constant related to the adsorption energy (dm^3^ g^−1^). The Langmuir isotherm model was found suitable to describe the phenol’s adsorption equilibrium (0.964 ≤ R ≤ 0.998). The efficiency of the adsorption process can be predicted by the dimensionless equilibrium parameter R_L_, which is defined by the following equation:

(7) $$R_{L}=\frac{1}{1+K_{L}C_{0}}$$

Here, C_0_ is the initial concentration of phenols in the solution (g dm^−3^).

The adsorption is irreversible when R_L_ = 0, favourable when 0 < R_L_ < 1, does not occur when R_L_ = 1, and unfavourable when R_L_ > 1. For our samples R_L_ values are always lower than 1 for all the substrates (the values are within 0.72–0.96, Table 3), which indicates a favorable adsorption.

The adsorption of I and II on S0 was clearly less favored than on S1 and S2. Doping with sulfur seemed to improve the adsorption of I and II on the catalyst surface. Compound III showed weak adsorption to all catalysts. 

### 3.2. Optical Energy Evaluations

Introducing sulfur into the TiO_2_ structure was expected to affect the electronic band structure in the examined photocatalysts. Thus, the band energies should be different in the doped and unmodified TiO_2_. Apart from knowing the band gap energy, the match and position of the conduction band (CB) and the valence band (VB) levels of a semiconductor photocatalyst with the redox potential of photocatalytic reactions were also very important. The conduction band energy (E_CB_) and the valence band energy (E_VB_) were related to the reduction potentials (E^0^, V) of e^−^ and h^+^ (respectively) arising on the surface of the catalyst upon optical excitation. Convenient comparison of E^0^_CB_ and E^0^_VB_ with the one-electron reduction potentials of organic compounds allowed the assessment of the potential photocatalyst efficacy in the pollutant reduction- and oxidation-type degradation. The E^0^ were calculated from the difference between band energy and Fermi energy (E_F_), since E_F_ is an equivalent of the zero potential vs. the SHE (normal or standard hydrogen electrode; from here on all reduction potentials will be presented vs. SHE).

Contrary to colourless anatase nanopowder the S-doped TiO_2_ nanopowders were yellow, immediately suggesting their visible-light absorption ability. The UV-Vis diffuse reflectance spectra of S0, S1, and S2 nanopowders are shown in Figure S1 (Supplementary materials). The S-doped catalysts exhibited stronger absorbance in the visible-light region than the commercial TiO_2_. However, there was no clear trend with the sulfur content discernible. Similar observations were made by Sraw et al. [32] and Piątkowska et al. [33] in their works.

The optical band gap (E_bg_) for samples was calculated from the Tauc plot with following equation:

(αhν) = A(hν − E_g_)^n^ (8)

**Figure 4:**
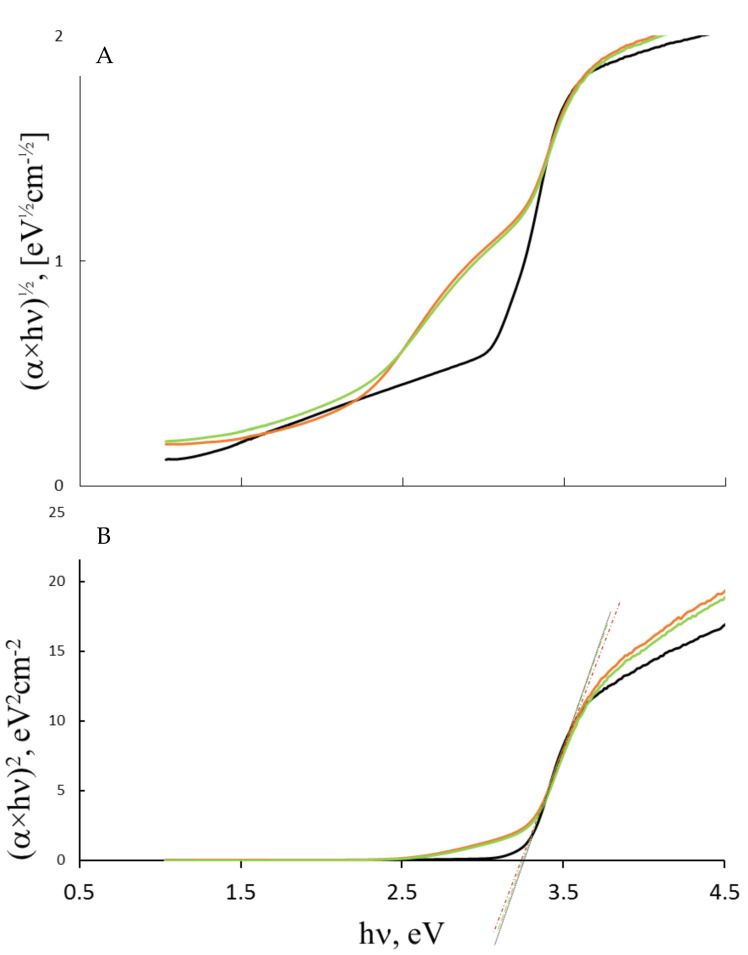
The Tauc plots of the catalysts particles; intra gap states (**A**); band gap estimation (**B**); black line represents commercial anatase (S0), green line represents S1 and red line S2.

Here, n is 0.5 and 2 for indirect and direct transitions, respectively [34]. In plots of (αhν)^n^ against hν, the optical band gap energy E_bg_ was determined by extrapolating the linear portion of the graph to (αhν)^2^ = 0 (Figure 4B).

The optical absorption edge in amorphous materials was characterized by the presence of an exponential tail. It can result from a structural disordering caused by impurity atoms, their chaotic distribution, and differences in average size. The absorption coefficient near the band edge showed an exponential dependence (8) on photon energy [35]:

(9) $$\alpha =\alpha_{0}\exp\left(\frac{h\nu }{E_{U}}\right)$$

where density of state is represented by the absorption coefficient (α) for given photon energy (hν). The α_0_—is the constant, whereas E_U_—denotes the Urbach energy which corresponded to the width of the band tail. Urbach energies were calculated as the reciprocal of the slope of the linear low energy part of the plot; ln(α) against photon energy (hν).

The impurity doping was the most commonly used technique for increasing optical absorption. Narrowing the band gap by introducing shallow impurity levels in the band gap, electron transition from the valence band to these impurity levels could be excited by low-energy photons. Moreover, since the special 3d^0^ configuration of Ti^4+^, the electronic structure of TiO_2_ could be easily modified by introducing new donor state in the band gap. As seen in Figure S1, there was a strong intrinsic absorption band which originated from the band gap excitation of electrons in TiO_2_ (it allows TiO_2_ to show only the photoresponse in the UV region). For S-doped TiO_2_, it was extended to the visible light region.

For semiconductors, the Urbach tail was related to the degree of crystal disorder and defects [35]. Samples with low levels of impurities, defects, and electron-phonon interactions tended to have small E_U_. The increasing trend in Eu values after doping was because of increasing disorders in the crystal lattice of S1 and S2. The lower Urbach energy indicates lower defect density in S0 catalyst.

The maximum absorbance wavelength was associated with the conduction band energy according to quantum theory. The photoenergy Equation (10)

(10) $$E_{\mathrm{cb}}=\frac{h\;\cdot \;c}{\lambda_{\max}}$$

allows calculation of the conduction band energy (E_CB_) from the UV-Vis absorption spectra (λ_max_—maximum absorbance wavelength, h—Planck’s constant, c—light speed in vacuum). The subtraction of E_bgd_ from the E_cb_ for respective catalysts gives the energy of its valence band (E_VB_). Using the derivative of the function Abs = f(λ), we found λ_max_ (98% edge saturation); hence, E_CB_ values were determined. 

When A_max_ (~E_bgd_) refers to the maximum absorption value in the UV-absorption edge, the E_F_ energy level can be calculated applying the Fermi–Dirac distribution function (11) [36],

(11) $$A_{\max}=\frac{1}{\left(1+\exp\left(\frac{E_{F}-h\nu }{k_{B}T}\right)\right)}$$

where hν is the photon energy in the absorption maximum, and k_B_ is the Boltzmann constant, and T is the absolute temperature. Plotting the function k (λ) shows that this function has absorption edges in the UV A range. 

The estimated values of E_U_, E_F_, E_CB_ and E_VB_, and reduction potentials E_CB_^0^ and E_VB_^0^ (vs. SHE) are presented in Table 4.

The sulfur incorporation introduces intra-gap states, which are thought to enhance the light absorption in the visible range. However, the sulfur doping of TiO_2_ only slightly narrowed the band gap. As the amount of sulfur dopant increases, the energy of the Fermi level increases. Similarly, the Urbach energy increases with the sulfur content. This is due to the fact that an increase in the number of dopants causes an increase in the number of crystal lattice defects. The determined values (E_bgd_, E_F_) allow one to estimate the value of the reduction potential of the conductivity band and the oxidative valence band. The electrochemical potentials of valence band and conduction band changed negligibly with sulfur content. 

The interfacial charge transfer efficiency is limited by two important processes: the competition between charge carrier recombination and trapping followed by the competition between trapped carrier recombination and interfacial charge transfer. One of the most important parameters that affect the efficiency of the electron transfer reactions is the driving force of the electron transfer reaction. The major parameter that affects the efficiency of the electron transfer reactions is the standard redox potential of the involved electron acceptor related to the standard redox potential of the conduction band electron (only those species with reduction potentials much more positive than the conduction band edge can be photoreduced). The E^0^_CB_ of catalysts were in the range −0.23 to −0.16 V. Thus, the electron potentials could not be negative enough to reduce dioxygen to superoxide O_2_^•−^ (E^0^ = −0.33 V [37]). After charge separation in the absence of suitable adsorbed hole scavengers, the remaining holes oxidise surface water to produce adsorbed hydroxyl radicals, which can subsequently induce further oxidation reactions. In alkaline solution, the reaction with hydroxyl anions is a source of hydroxyl radicals (E^0^(HO^•^/OH^−^) = 1.9 [38]), which can react with the compounds on the surface and in bulk of the solution. E^0^_VB_ catalysts was determined to be from 3.03 to 3.06 V (vs. SHE) (Table 3). This suggests that hydroxyl radicals could be formed on catalysts surface, and additionally compounds I, II, and III could undergo direct oxidation by photo-generated holes. In alkaline media, the ^–^OH groups suffer deprotonation, which caused a pH-independent oxidation that occurred at a lower potential. The phenolates (i.e., the phenolate anions) are much easier to be oxidized than phenols, and, for pH > pK_a_, their oxidation is favored. BPA oxidation at the Pt electrode was reported by Tanaka et al. in the range pH 2–12 [39]. At pH = 10 they observed two peaks due to pK_a_ of bisphenol A. The two anodic waves corresponding to the oxidation of two species—neutral bisphenol A (ca. 0.912 V vs. SHE) and phenolate ion (ca. 0.582 V vs. SHE). It means that the anions are oxidized easier and that the hole generated on the catalysts has enough positive potential for direct oxidation of II.

### 3.3. Photocatalytic Activity

**Figure 5:**
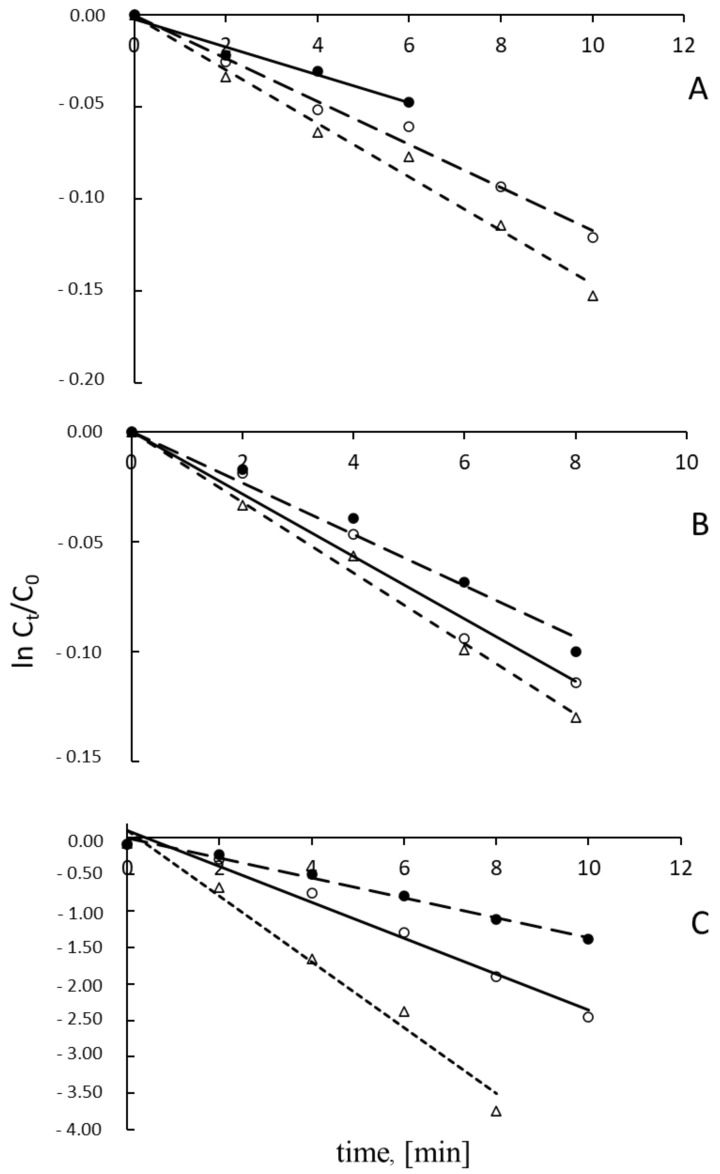
First-order plot of the relative concentrations (C/C_0_) vs. time, obtained upon illumination of air-saturated aqueous solutions, pH 10, 2.0 × 10^−4^ mol dm^−3^ of the test-compounds: I (**A**), II (**B**), and III (**C**) with suspended particles of the photocatalysts. The filled circle (●) represents the compounds in the S0-catalyst suspensions, the open circle (o) in the S1-catalyst suspension, while open in the triangle (∆) S2-catalyst suspensions.

A plot of ln(C/C_0_) versus time yields a straight line where k_app_ is the slope (see Figure 5).

Photocatalytic activities of the catalysts were estimated by monitoring decomposition of test-compounds in the catalyst suspensions upon illumination. The obtained data are presented in Figure 5, whereas the first decay half-lives (t_1/2_) and the apparent rate constants (k_app_) calculated from the plots are summarized in the Table 5. 

The photocatalytic activity tests were performed in alkaline aqueous solution (pH 10). Under such conditions the phenols are present in solution in dissociated and undissociated form—the respective first and second acid dissociation constants, pK_a_, are 9.994 for I [40]; 9.59 and 10.2 for II [41] and 7.5 and 9.5 for III [38,42]. The simulated (using pK_a_ of compounds and Curtipot software [43]) molar fraction of ionic forms at pH 10 was: TBBPA^2–^ 0.869, TBBPA^–^ 0.131, TBBPA 0.00, BPA^2–^ 0.503, BPA^–^ 0.381, BPA 0.116, PhO^–^ 0.565, PhOH 0.435. Almost over the entire time region, the substrate decays obeyed the pseudofirst-order kinetics Equation (12):

(12) $$\ln\frac{C_{t}}{C_{0}}=-k_{\mathrm{app}}t$$

The phenols degradation rates increase with increasing of surface charge density and zeta potential value. Phenolate ions and hydroxyl ions compete for acid sites on the catalyst surface. The best adsorption for I and II was observed on the S2 catalyst. Notably, this finding coincides with the highest degradation efficiency of these compounds. This is due to the greatest number of acid sites on the surface (positively charged) of this catalyst, which can interact with negative charged phenolate ions. Close to one half of the molecules of I were undissociated at the given pH. Due to this fact, it may be caught by basic sites on catalysts surface. In the case of compound II, almost 90% was in the dissociated form. Accordingly, adsorption of II was only sluggish on the S0 catalyst (the most negative value of the surface charge), higher for the S1 and S2 catalysts—similar for both. This is reflected in the k_app_ values—the degradation rate II on the catalysts S1 and S2 was almost the same. High concentrations of hydroxyl ions caused a competition of phenolate ions and hydroxyl ions for acid sites on the surface of catalysts S1 and S2. However, this did not reduce the efficiency of oxidative degradation since I and II can degrade both in direct reaction with the hole and in reaction with hydroxyl radical. The compound III exists entirely in dissociated form. Hence, it did not adsorb on the catalysts surface. Surprisingly, however, the observed k_app_ of degradation for III was the highest among the tested compounds. The degradation rate of III varied in the series S0 < S1 < S2. The highest degradation rate was observed for the catalyst having the most acid sites. These results do not correlate with the measurements of adsorption III on the catalysts.

The degradation rate of I varied in the series S0 < S1 < S2. The lowest degradation rate of I on S0 catalysts may be caused by low adsorption on its surface. The lowest rate of I degradation on the S0 catalyst may be due to the low adsorption. The S0 catalyst had the lowest specific surface area. Additionally, S0 had the most negative surface charge of all catalysts, so the PhO^−^ ions would be repelled by the catalyst surface. Only undissociated phenol molecules approached the catalyst surface (Coulomb interactions) and reacted. The high activity of catalysts S1 and S2 in the phenol degradation may be ascribed to two factors. The S-TiO_2_ catalysts have small particle sizes and large specific surface areas (Table 1), which reduces the path length for charge carriers diffused from the bulk to the surface of TiO_2_, lessening the volume recombination probability of the charge carriers. Moreover, a shift of the absorption edge toward the visible light regions of the solar spectrum was observed for S-TiO_2_. The irradiation of S1 and S2 produced more excited electrons (e^−^) and holes (h^+^), probably due to the presence of doped sulfur that avoided charge recombination, improving photocatalytic activity. With the increasing sulfur doping, the photocatalytic degradation rate of phenol increased (Figure 5A). 

The apparent reaction constant (k_app_) shows that, in all cases, S2 catalyst (the most sulfur-loaded) exhibited the best photocatalytic activity; however, catalysts S1, and S2 were equally effective for the degradation of II.

The rate constants of degradation reactions (k_app_) value increased as the catalyst particle size decreased. Specific surface area increased with decreasing catalysts particle size. The hydrodynamic radius of the catalyst (observed under the reaction conditions) decreased with increasing catalysts grain diameter. As the hydrodynamic radius increased, the surface charge of the catalyst increased, and the degradation reaction rate increased in line with this trend. 

Considering the adsorption equilibrium constant and degradation apparent rate constant, we conclude that photocatalytic efficiency was related with the catalysts surface-substrate solution interactions. Presence of I in the anionic form favoured the adsorption at the S1 and S2 catalyst surface, causing a higher rate of degradation than that observed for S0. 

The adsorption of II under the reaction conditions strongly depended on the surface charge of the catalyst (50% of II is completely dissociated; catalysts surface charge was negative). BPA adsorbs best onto S2, although the rates of degradation reactions onto S1 and S2 are comparable. Since it is difficult for BPA to be adsorbed on the TiO_2_ surface at alkaline pHs, BPA would have a reduced chance to react with the photogenerated hole on the TiO_2_ surface and tended to react with the diffused hydroxyl radicals in the bulk liquid. This effect may be attributed to more efficient generation of hydroxyl radicals (^•^OH) on the TiO_2_ surface, with an increase of hydroxide ion (OH^−^) concentration. Therefore, in this case, the presence of large quantities of OH^−^ on the TiO_2_ particle surface may have favoured the production of hydroxyl free radicals and improved the BPA photocatalytic degradation efficiency.

Chemically, the surface condition of the catalyst can be represented as a solid/solution interface partially co-ordinated with an organic compound. The substrates bonding to catalysts surfaces may be realised via electrostatic interactions [44]:

(13) $$S\equiv O^{-}+\mathrm{Ph}-\mathrm{OH}⇋S\equiv O^{-}\cdots \mathrm{HO}-\mathrm{Ph}$$

(14) $$S\equiv O^{-}+R-\mathrm{Ph}-\mathrm{OH}⇋S\equiv O^{-}\cdots \mathrm{HO}-\mathrm{Ph}-R$$

**Figure 6:**
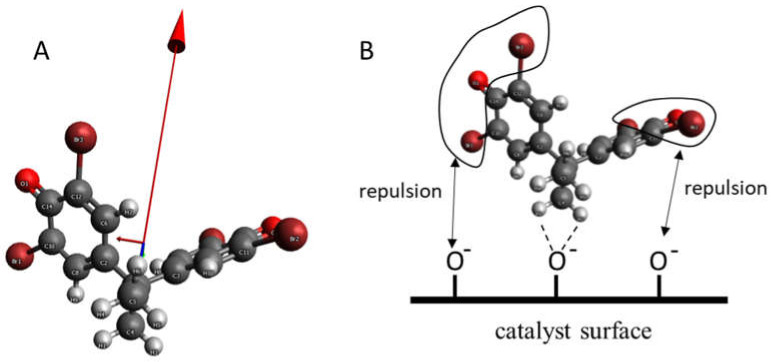
(**A**) Dipole moment direction in compound III (direction of the moment is marked with the arrow); (**B**) catalyst/TBBPA interactions under reaction conditions.

**Figure 7:**
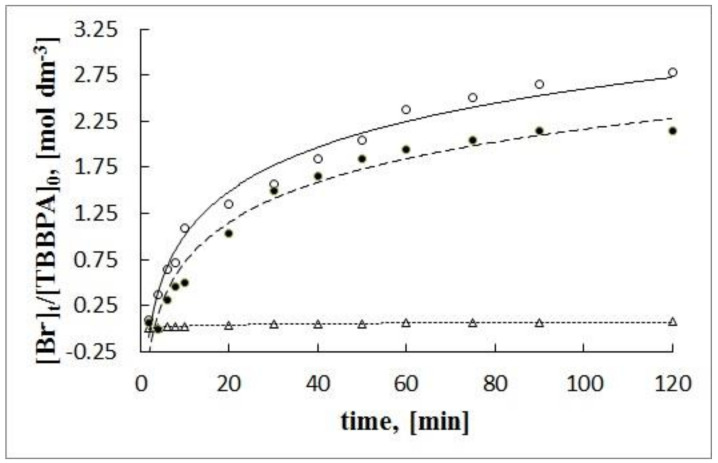
Relative concentrations of Br^−^ vs. time profile obtained upon illumination of air-saturated aqueous suspensions, pH 10, 2.0 × 10^−4^ mol dm^−3^ of substrate III. The filled circle (●) represents the compounds in the S0-catalyst suspensions, the open circle (o) in the S1-catalyst suspension, while open triangle (∆) in the S2-catalyst suspensions.

Compound III was completely deprotonated at pH 10, so it is repelled by Coulombic forces by the negatively charged catalysts surface and its adsorption was hindered. However, it could approach the catalyst surface through the methyl groups, as is shown in Figure 6. Quantum-chemical calculation allowed us to determine the value (ca. 8.21 Debye) and direction of the dipole moment of ion III (Symbolized by the red arrow in Figure 6A), which explains the interaction of anion III with the negatively catalyst surface presented in Figure 6B. The weak adsorption of III should affect negatively the photodegradation; however, its rate of degradation was the best among tested compounds. It is probably caused by bromine substituents in the benzene ring, which allows a reductive degradation mechanism. The pronounced differences in apparent decomposition rates, shown in Figure 5C and Figure 7, could not be fully explained in terms of the oxidation mechanism being the only pathway. The observation shown in Figure 5C points to a superposition of (i) one-electron oxidation followed by debromination and further fragmentation, and (ii) dissociative electron transfer resulting in fast debromination. Electron-transfer induced dehalogenation of organic compounds is a common mechanism in their redox chemistry [45]. There were instances where transfer of an electron to a neutral precursor leaves the resulting radical ion in an electronic ground state that is dissociative. The process is called dissociative attachment [46]: Following the electron-transfer event, which is rapid on the time scale of nuclear motion, the ion relaxed along the dissociative coordinate, leading to the scission of one or more bonds. It is believed that, for aromatic halides, the reductive cleavage occurred in a two-step mechanism (RX + e–→ RX^•–^→R^•^ + X^–^), with the transient formation of a radical anion (RX^•–^). In particular, the massively accelerated degradation of III indicated reductive debromination to be very efficient.

The degradation reaction rate constant for compound III was the highest on catalyst S2, whereas, on S0, it was the lowest. The situation was quite different when considering the rate of bromide ion release (Figure 7). The yield of bromide ion formation during the degradation reaction of III (S1 > S0 > S2) corresponded to changes in the adsorption’s value equilibrium constant of compound III on the catalysts. The condition for the course of the reduction process was the adsorption of the substrate on the catalyst. Horikoshi et al. [47] have suggested the formation of bromide ions from TBBPA degradation in UV irradiated alkaline aqueous TiO_2_ dispersions. However, the release of Br^−^ can be caused by both the reductive debromination and hydroxyl radical oxidation. 

Photodegradation of phenols was extensively studied since the advent of the method. Ever since numerous experiments provided very interesting but scattered data. Since the experimental conditions varied between particular experiments seems that only one parameter, namely apparent τ_1/2_, could be utilized in order to somehow compare the efficacy of degradation. 

For example, early mechanistically oriented works, which concentrated on pristine TiO_2_ gave for phenol very unsatisfactory τ_1/2_ equal ca. 8000 h Sobczyński et al. [48]. Whereas, the recent usage of catalyst formulated based on Cu-doped NiO pushed the τ_1/2_ of phenol degradation below two hours Ethiraj et al. [49]. Similarly, a recently published study on a new composite catalyst containing Kaolinite, cement, and wood fibers modified by titanium oxide shows shortening of phenol degradation τ_1/2_ to 75 min Morjene et al. [50].

In recent work Pei et al. [51] report usage of fluorine-doped titanium suboxide anode (F-doped TiSO) for electrochemical degradation of TBBPA. The τ_1/2_ obtained there, equal ca. 4 min, is comparable with our results (present study), which is in the range of 1.5–5.3 min.

Previous, work of Horikoshi et al. [47] where degradation of BPA and TBBPA in UV-irradiated alkaline aqueous TiO_2_ dispersions (pH = 12) where studied shows for BPA and TBBPA degradation τ_1/2_ ca. 32 min and 26 min, respectively. These results outperform our results for BPA but not for TBBPA.

## 4. Conclusions

The sulfur-doping of TiO_2_-based catalysts increased their photocatalytic activity, thus accelerating degradation of investigated organic pollutants in water. A positive effect was observed for both oxidative and reductive degradation pathways. The reductive degradation pathway is sensitive to the amount of sulfur-dopant in a non-monotonous manner: higher doping (S2 catalyst) seemed to accelerate an electron-hole recombination impairing reduction. The studies reported in this paper have shown that S1 catalyst was the most efficient in photoreduction process of III, which was highly susceptible to reductive attack, concomitant with bromide expulsion. S1 has the smallest indirect band gap, which renders the material responsive towards visible light and electron-hole pair production at low light energy. In this system, the recombination lifetime lengthens compared to direct band gap. Accordingly, the probability of reaction between electron and the adsorbed organic substrate on photocatalyst surface increases. 

The divergent photocatalytic degradation efficiency of I, II, and III can be ascribed to the catalyst’s surface-substrate solution interactions (e.g., K_L_). The yield of bromide ion formation during the degradation reaction of III (S1 > S0 > S2) corresponds to changes in the adsorption’s value equilibrium constant of compound III on the catalysts. The major condition for the course of the reduction process was the adsorption of the substrate on the catalyst. Adsorption of substrates on the surface of the catalyst was the main factor influencing the rate of their degradation, especially for compound III. The differences in the bromide ion release profiles result from the different participation of the oxidation and reduction processes in the degradation reaction. The other parameter which effects on bromide ions formation efficiency may be the sulfur content in catalyst. Higher sulfur content in S1 can trap electrons at energy levels corresponding to sulfur and thus reduce the effectiveness of the reductive process.

## Figures and Tables

**Table 1:** Catalyst particle parameters.

Sample	ξ-Potential ^#^ [mV]	Hydrodynamic Diameter ^#^ [nm]	Đ ^#^	Surface Area, m^2^g^−1^	Size [nm] XRD	Sulfur Content [atom%] ^##^
**S0**	−52.17 ± 1.01	340.00	0.22	8.90	46.00	0
**S1**	−36.64 ± 0.55	416.00	0.24	52.30	20.00	0.15 ± 0.05
**S2**	−34.73 ± 0.47	504.00	0.27	55.70	18.50	0.36 ± 0.04

^#^ suspension concentration 6.25 × 10^−4^ g cm^−1^, pH = 10. ^##^ EDS measurements.

**Table 2:** Catalysts’ surface properties.

Sample	pK_1_	pK_2_	pH_pzc_	Surface Charge Density [C m^−2^] pH = 10	ζ Potential [mV] pH = 10
**S0**	2.51 ± 0.2	9.63 ± 0.2	6.07 ± 0.1	−321.00	−52.17 ± 1.01
**S1**	2.39 ± 0.2	11.28 ± 0.2	6.84 ± 0.1	−59.50	−36.64 ± 0.55
**S2**	2.61 ± 0.2	11.30 ± 0.2	6.96 ± 0.1	−53.00	−34.73 ± 0.47

**Table 3:** Adsorption parameters of substrate-catalyst systems.

Substrate/Catalyst	C_0_ [g dm^−3^]/[mol dm^−3^]	K_L_ [dm^3^ g^−1^]	Q_max_ [g g^−1^]/[mol g^−1^]	R_L_
**I**	1.88 × 10^−2^/2.00 × 10^−4^			
**S0**		3.74	2.28 × 10^−2^/2.42 × 10^−4^	0.93
**Ca.**		7.47	1.00 × 10^−2^/1.07 × 10^−4^	0.88
**S2**		6.59	1.33 × 10^−2^/1.41 × 10^−4^	0.89
**II**	4.57 × 10^−2^/2.00 × 10^−4^			
**S0**		1.01	19.91 × 10^−2^/8.72 × 10^−4^	0.96
**S1**		7.27	6.11 × 10^−2^/2.67 × 10^−4^	0.75
**S2**		8.30	9.23 × 10^−2^/4.04 × 10^−4^	0.72
**III**	1.10 × 10^−2^/2.00 × 10^−4^			
**S0**		3.10	8.96 × 10^−2^/1.65 × 10^−4^	0.75
**S1**		3.72	4.07 × 10^−2^/0.75 × 10^−4^	0.71
**S2**		0.89	12.13 × 10^−2^/2.23 × 10^−4^	0.91

**Table 4:** Optical energies of the catalysts.

Sample	XRD Size, nm	E_bgd_^a^, eV	E_bgi_^b^, eV	E intra Gap State [eV]	Eu, eV	E_F_, eV	E_CB_, eV	E^0^_CB_, V vs. SHE	E_VB_, eV	E^0^_VB_, V vs. SHE
**S0**	46.0	3.26	2.86	-	0.27	3.46	3.69	−0.23	0.43	+3.03
**S1**	20.0	3.24	2.68	1.81	0.32	3.59	3.77	−0.16	0.53	+3.06
**S2**	18.5	3.22	2.64	1.89	0.33	3.60	3.78	−0.18	0.56	+3.04

^a^ direct band gap energy. ^b^ indirect band gap energy.

**Table 5:** Kinetic data.

Compounds ⇒	I		II		III	
Photocatalyst	t_1/2_, min	k_app_ × 10^−3^, min^−1^	t_1/2_, min	k_app_ × 10^−3^, min^−1^	t_1/2_, min	k_app_ × 10^−3^, min^−1^
**S0**	91.20	7.60	59.24	11.70	5.28	131.40
**S1**	59.24	11.70	49.16	14.10	2.99	231.40
**S2**	47.48	14.60	43.05	16.10	1.54	450.10

## Data Availability

The data presented in this study are available on request from the corresponding author. The data are not publicly available because they are part of the ongoing project.

## Supplementary Materials

The following are available online at https://www.mdpi.com/article/10.3390/ma15010361/s1, Figure S1: The UV-Vis diffuse reflectance spectra of catalysts, Figure S2: The UV-Vis absorbance spectra of I, II, III, Figure S3: EDS spectrum of S1 (A) and S2 (B), Figure S4: Surface charge density of studied catalysts The filled circle (●) represents the compounds in the S0-catalyst suspensions, the open circle (o) in the S1-catalyst suspension, while in the open triangle (∆) S2-catalyst suspensions, Figure S5 Substrate adsorption as surface charge density function.
