# Supplementary material for: Photocatalytic Degradation of 4,4′-Isopropylidenebis(2,6-dibromophenol) on Sulfur-Doped Nano TiO2
Source: Materials (Basel). 2022 Jan 4;15(1):361. doi: 10.3390/ma15010361 (PMC8746070; doi:10.3390/ma15010361)
Supplement: Supplementary file 1 [file materials-15-00361-s001.zip › materials-1466384-supplementary.pdf]

materials

Photocatalytic Degradation of 4,4′-Isopropylidenebis (2,6-dibromophenol) on Sulfur-Doped Nano TiO_2

Joanna B. Kisała [1,*], Gerald Hörner [2], Adriana Barylyak [3], Dariusz Pogocki [1,4] and Yaroslav Bobitski [1,5]

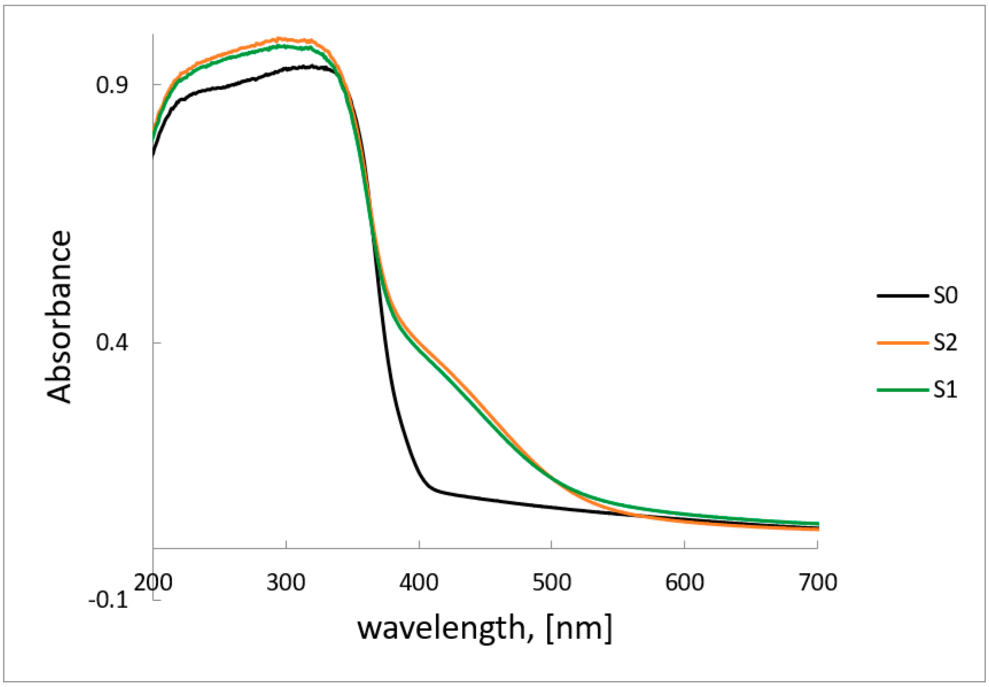

Figure S1. The UV-Vis diffuse reflectance spectra of catalysts.

Citation: Kisała, J.B.; Barylyak, A.; Pogocki, D.; Bobitski, Y. Photocatalytic Degradation of 4,4′-Isopropylidenebis(2,6-dibromophenol) on Sulfur-Doped Nano TiO_2. *Materials* **2022**, *15*, 361. https://doi.org/10.3390/ma15010361

Publisher's Note: MDPI stays neutral with regard to jurisdictional claims in published maps and institutional affiliations.

Copyright: © 2022 by the authors. Licensee MDPI, Basel, Switzerland. This article is an open access article distributed under the terms and conditions of the Creative Commons Attribution (CC BY) license (http://creativecommons.org/licenses/by/4.0/).

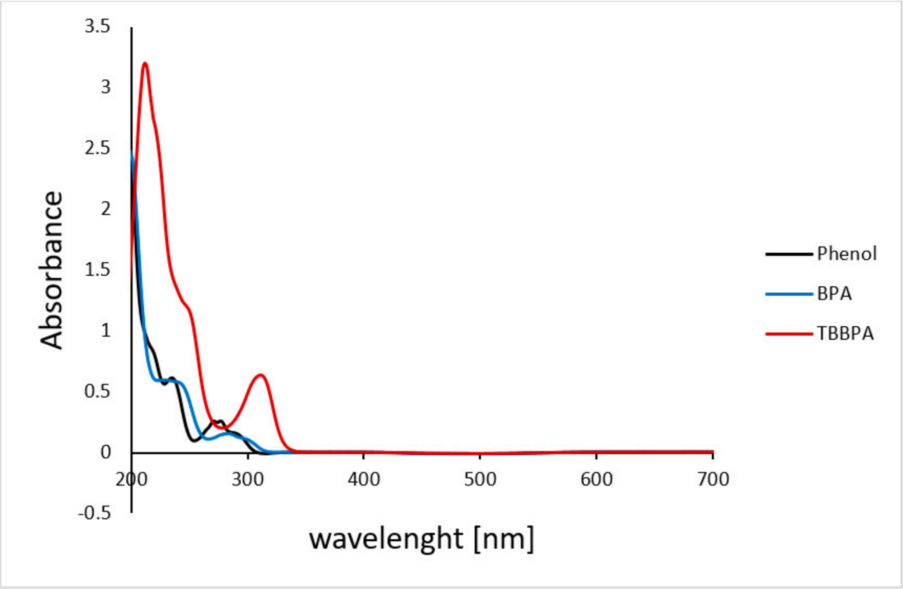

Figure S2. The UV-Vis absorbance spectra of **I**, **II**, **III.**

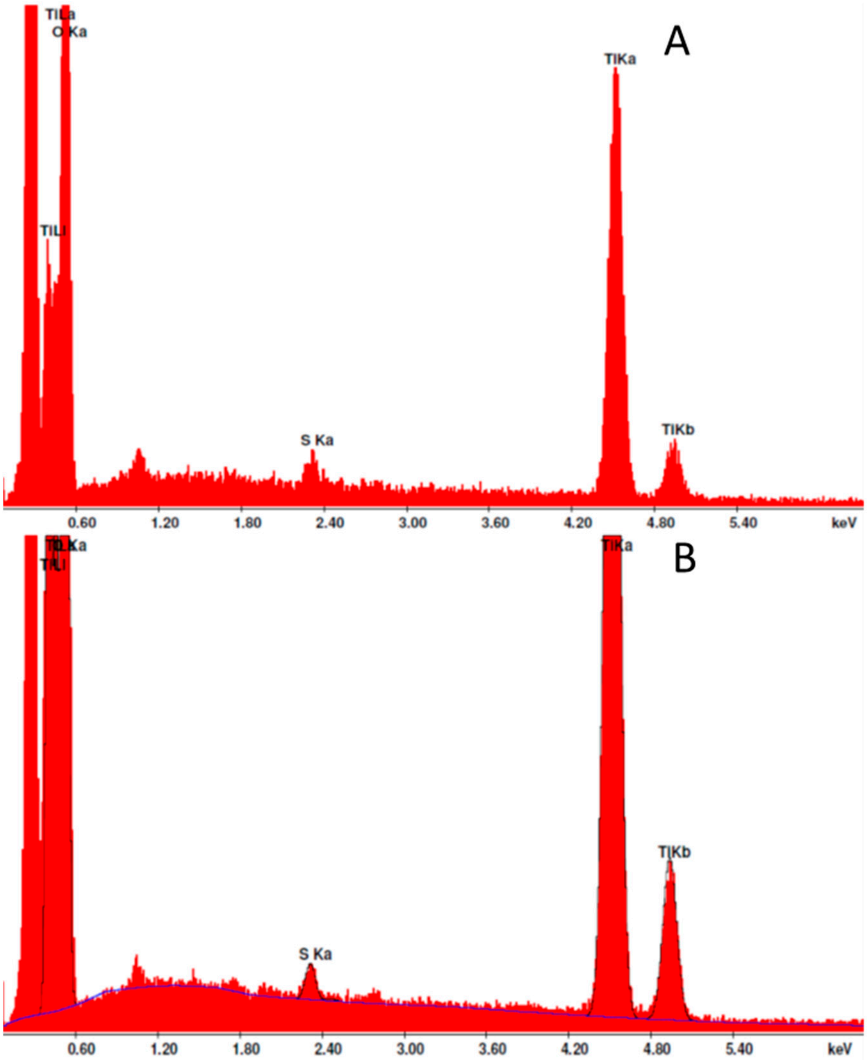

Figure S3. EDS spectrum of S1 (**A**), and S2 (**B**).

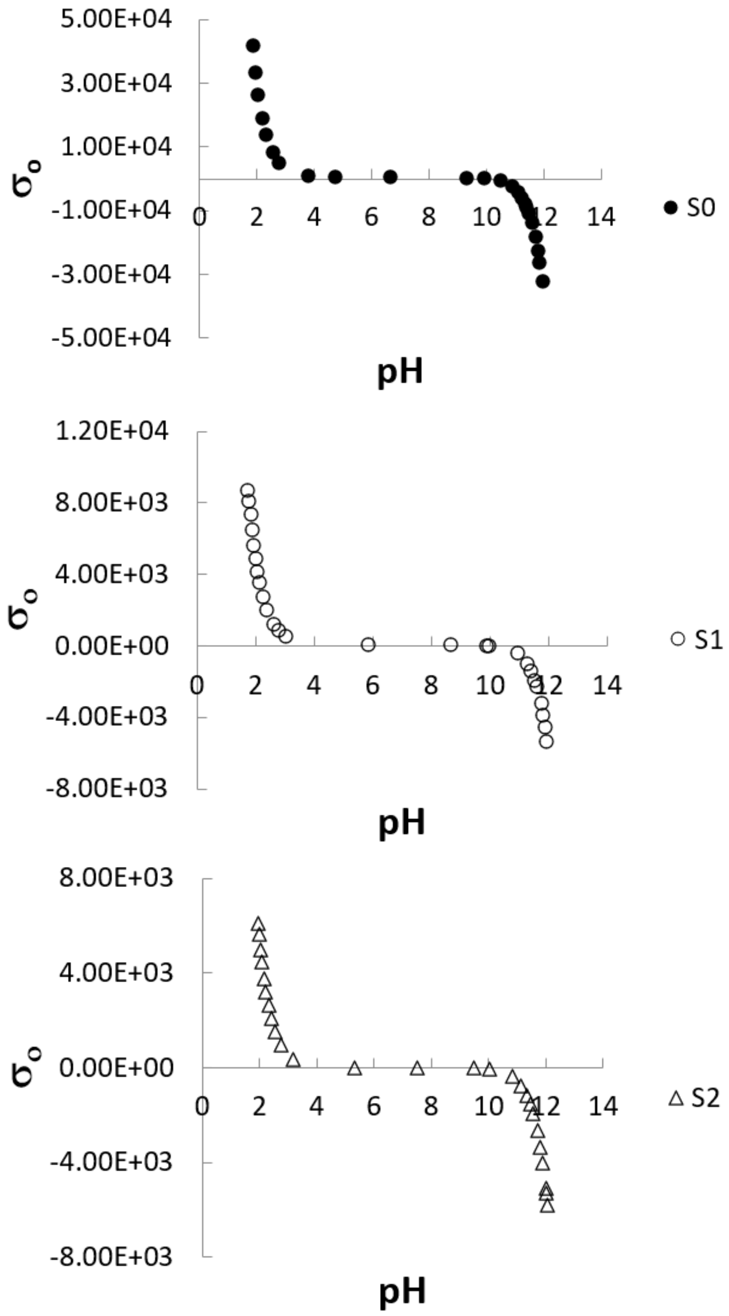

Figure S4. Surface charge density of studied catalysts The filled circle (●) represents the compounds in the S0-catalyst suspensions, the open circle (o) in the S1-catalyst suspension, while in the open triangle (Δ) S2-catalyst suspensions.

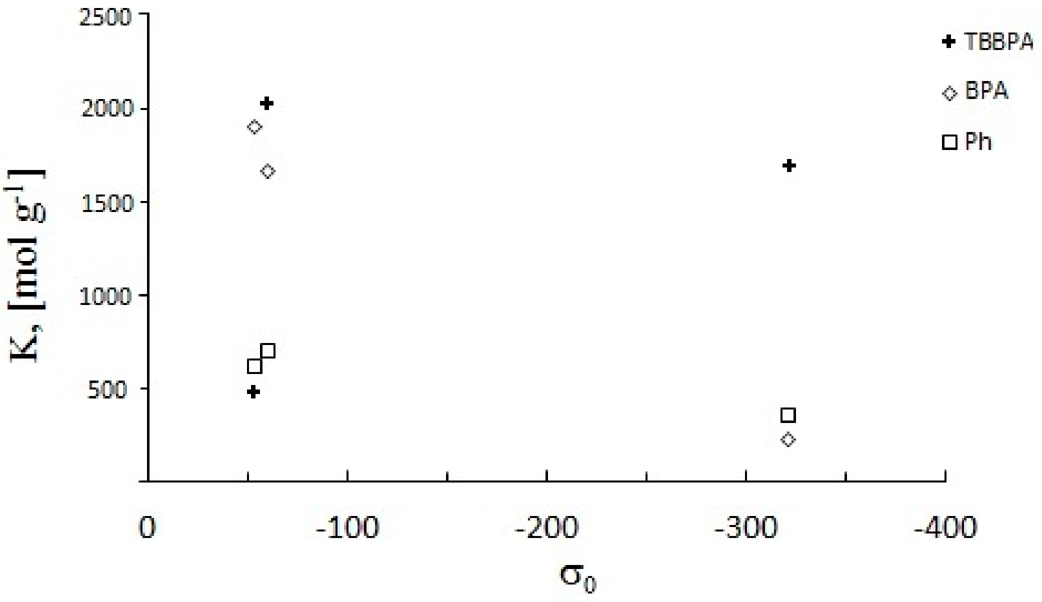

Figure S5. Substrates adsorption as surface charge density function.
